# Keratoacanthoma centrifugum marginatum: Clinical and dermoscopic presentation and considerations on spontaneous involution

**DOI:** 10.1016/j.jdcr.2026.02.010

**Published:** 2026-02-12

**Authors:** Esther Verónica Echeverry Rodríguez, Carolina Concha Arango, Luis Fernando Cárdenas Cardona, Edgar Andrés Lozano Ponce

**Affiliations:** a Department of Internal Medicine, Dermatology, School of Health, Universidad del Valle, Cali, Colombia; b Dermatology Section, University Hospital of Valle, Cali, Colombia

**Keywords:** dermoscopy, keratoacanthoma centrifugum marginatum, skin of color, spontaneous regresión, squamous cell carcinoma differential diagnosis

## Introduction

Among keratoacanthomas, keratoacanthoma centrifugum marginatum (KCM) is a rare and infrequent presentation. It exhibits progressive surrounding growth, leaving central scarring and atrophy as it expands peripherally. It grows to a large size, sharing clinical similarities with squamous cell carcinoma, and its diagnosis is often difficult. Dermoscopic descriptions of KCM are scarce, and spontaneous regression remains a matter of debate, occurring in only a few cases. We present a case of KCM in a patient, who developed distinctive dermoscopic features with partial spontaneous involution, contributing to the understanding of the clinical and biological behavior of this tumor.

## Case report

**Fig 1:**
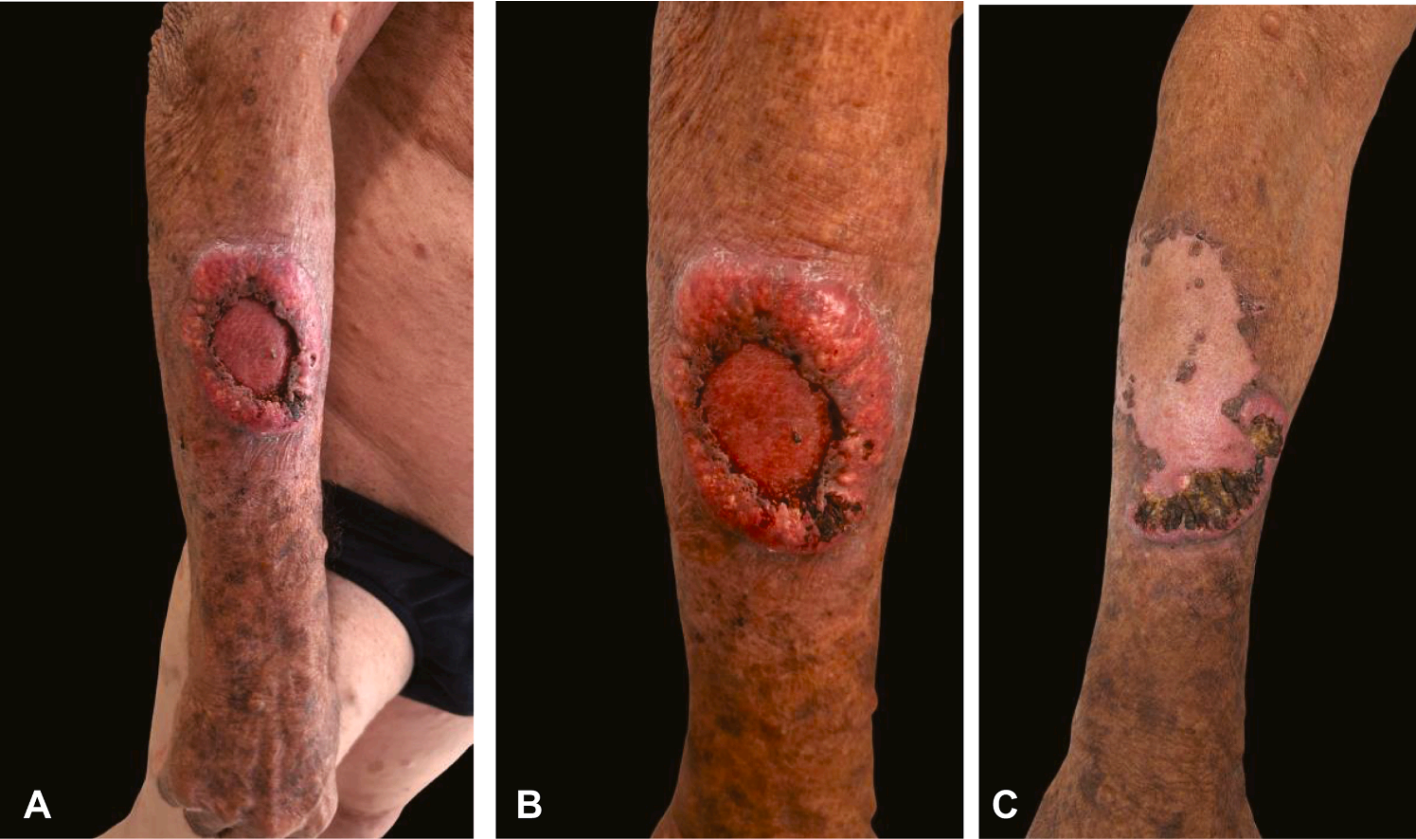
Clinical presentation. **A,** Well-demarcated tumor plaque, 9 × 15 cm, located on the posterior aspect of the right forearm, erythematous with a central crater with a scar-like appearance, thick rolled edges with whitish globules, and a scalloped edge. **B,** Close-up, note the scalloped edge with microulcerations. **C,** Six months later, regression of the lesion and centrifugal spread in the lower part.

A 66-year-old man from Nariño (Colombia) presented a lesion on his right forearm, which occurred more than 2 months after trauma from thorn. The lesion had progressively increased in size. Physical examination revealed a well-demarcated, oval tumor plaque measuring approximately 15 × 9 cm with highly raised margins, a foul odor, and a scar-like central crater (Fig 1, *A* and *B*).

**Fig 2:**
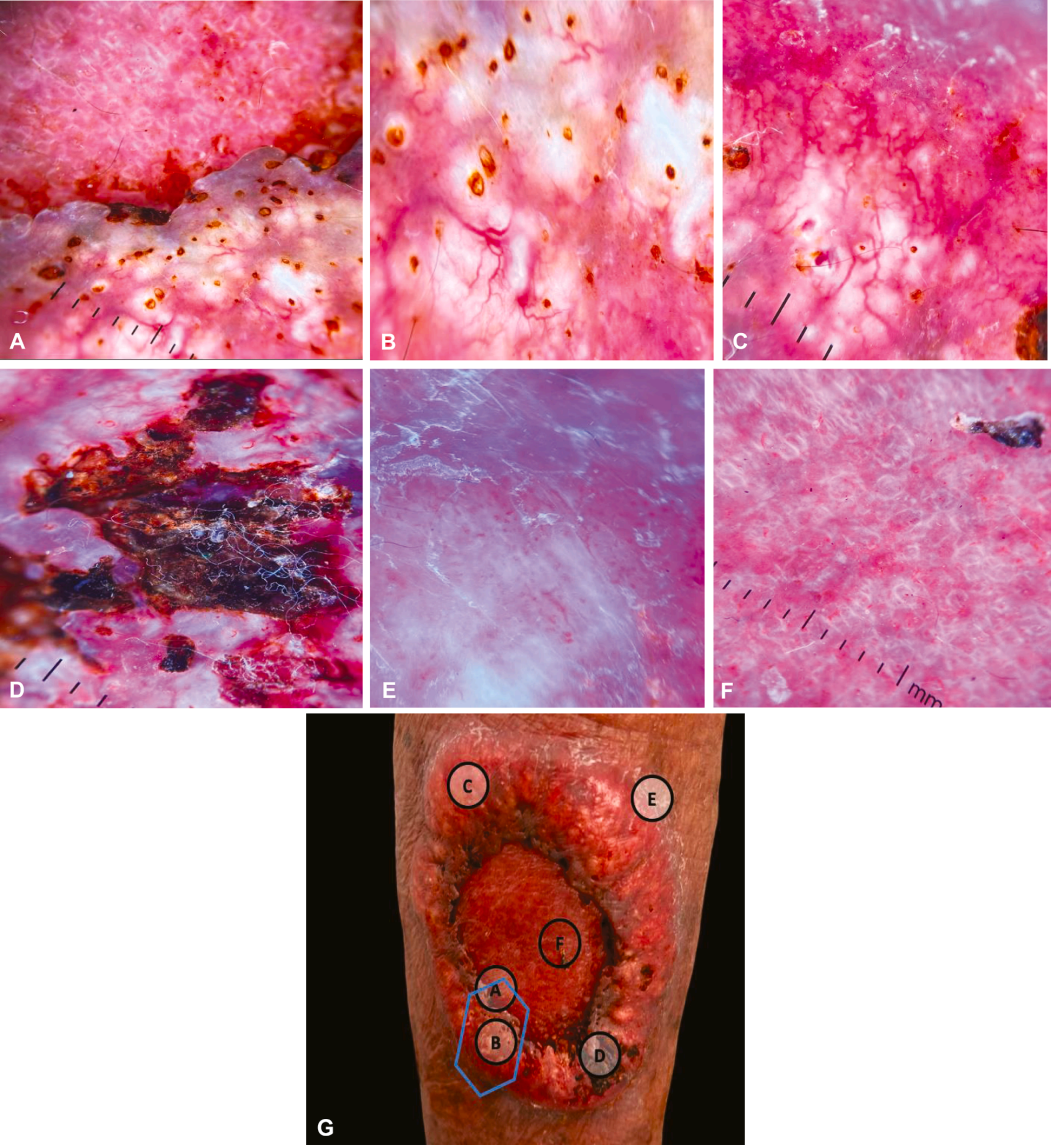
Dermoscopy. **A,** The raised edge shows a *pink* background with masses and lumps of *yellowish-white* keratin. Ocher-colored microulcerations and a circular fenestrated edge, with hemorrhagic crusts surrounding a central crater. Thick arborizing vessels are visible. **B** and **C,** Short, fine telangiectasias in focus and a superficial vascular network. **D,** Areas of hemorrhage, with ocher and *brown crusts*, thread sign. **E,** Structureless *white* and *milky-red* areas with punctate and glomerular vessels. **F,** Center of the lesion: *pink* background with *bright white* structures in a circular and linear pattern. **G**, Dermoscopic image locations A to F (*black circles*). The incisional biopsy location is marked in *blue*.

Dermoscopy performed in different areas of the tumor revealed a pinkish central background, bright white circular structures around the follicular openings, and a violet pigmentation along the raised margin, along with yellowish-white keratinous aggregates, birefringent white areas, ochre-colored ulcerations, hemorrhagic crusts, and a polymorphic vascular pattern consisting of thick and thin arborizing vessels and comma-shaped, punctate vessels and a homogeneous vascular network (Fig 2).

**Fig 3:**
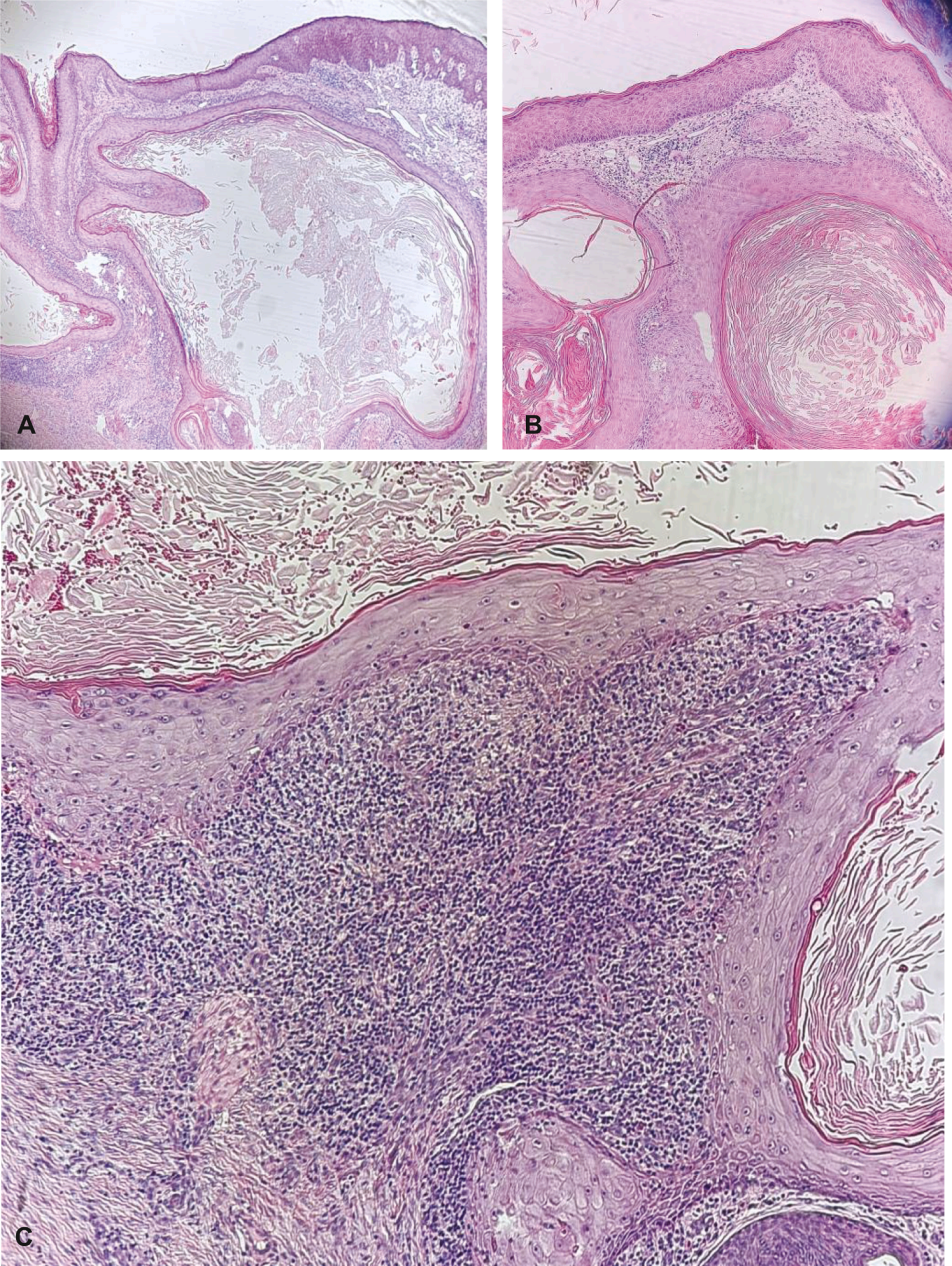
Histopathology. **A,** 4× invaginating pseudoepitheliomatous hyperplasia with a keratin-filled crater and pronounced reactive fibrosis. **B,** 4× large, *ovoid cells* with extensive cytoplasm, conspicuous and prominent nucleoli, dyskeratinocytes, and abundant keratin pearl formation arranged in invasive flaps. **C,** 10× dense band-like lymphohistiocytic infiltration with a lichenoid appearance in the dermis.

An incisional skin biopsy was performed from the edge to the center of the lesion, and histopathology revealed a keratoacanthoma, with well-differentiated keratinocytes invading the papillary dermis, with a cleft between the epithelium and the underlying dermis, containing a scar-like stroma surrounded by an inflammatory lichenoid-type inflammatory infiltrate (Fig 3). Returned for follow-up in 6 months, a delay due to nonrelated issues. The lesion showed partial spontaneous regression, with centrifugal displacement of the lower portion (Fig 1, *C*).

A diagnosis of KCM was made based on clinicopathological correlation, as it represented a lesion characterized by slow peripheral growth, a raised border, and an atrophic cicatricial center, located in a photoexposed area such as the forearm, along with a well-differentiated epithelial proliferation on histopathology, showing keratinocytes with minimal atypia and a dermal inflammatory infiltrate. Unlike typical keratoacanthoma, spontaneous regression is incomplete, which justifies its management as a locally aggressive lesion. Therefore, the patient was referred to oncological surgery for resection of the residual tumor, a computed tomography scan was ordered, and the patient was referred to another institution, resulting in loss of follow-up.

## Discussion

KCM is a very rare and uncommon presentation of keratoacanthoma. It is characterized by progressive peripheral growth that leads to central scarring or atrophy. Lesions can reach large sizes, with reports of up to 20 to 30 cm, so the differential diagnosis in the initial clinical evaluation is based on aggressive neoplasms, such as squamous cell carcinoma. Regarding its etiology, ultraviolet radiation, mineral oils, chemical carcinogens, and trauma (as in this case) have been reported.^1^^,^^2^

Dermoscopy reports are scarce given the low frequency of its presentation,^3^^,^^4^ which show characteristics that partially overlap with our observation. However, in our case, we identified novel features such as bright white circular structures, birefringent areas (possibly representing microcalcifications or keratin), and a “thread sign,” along with ulcerations, crusts, and polymorphic vascular structures. The size of the lesion allowed for dermoscopy of the central area, which presented a pink background with bright white structures in a circular and linear pattern (Fig 2). The bright white structures within the crater could correspond to fibrosis or granulation in dermal papillae derived from scarring, while the necrotic areas could herald spontaneous involution. Although spontaneous regression of KCM is extremely rare, there are documented cases^5^^,^^6^ and it deserves to be considered during the clinical evaluation. Nevertheless, the treatment of choice is usually surgical excision or administration of systemic retinoids, with favorable results in many cases.^7^ Responses to topical 5-fluorouracil and systemic or intralesional methotrexate combined with corticosteroids have also been described.^8^^,^^9^

Spontaneous regression in keratinocyte carcinomas has been attributed to a retinoic acid-mediated shift toward keratinocyte differentiation with Wnt inhibition, coupled with an immunologic response that promotes tumor involution; however, because of its histoclinical similarity to squamous cell carcinoma and the potential for extensive involvement, the approach remains excision with clear surgical margins or Mohs surgery when possible, reserving systemic/topical retinoids, intralesional agents, and other modalities for inoperable cases, and leaving observation for highly selected situations with close follow-up.^10^

KCM remains a rare and uncommon entity, and regression is observed very infrequently. We present a dermoscopic characterization of this KCM variant and describe new dermoscopic clues that could aid in the differential diagnosis.

## Conflicts of interest

None disclosed.
